# Nutritive and Bioactive Properties of Mesquite (*Prosopis pallida*) Flour and Its Technological Performance in Breadmaking

**DOI:** 10.3390/foods9050597

**Published:** 2020-05-07

**Authors:** Ursula Gonzales-Barron, Rody Dijkshoorn, Maikel Maloncy, Tiane Finimundy, Ricardo C. Calhelha, Carla Pereira, Dejan Stojković, Marina Soković, Isabel C. F. R. Ferreira, Lillian Barros, Vasco Cadavez

**Affiliations:** 1 Centro de Investigação de Montanha (CIMO), Instituto Politécnico de Bragança, Campus de Santa Apolónia, 5300-253 Bragança, Portugal; tiane@ipb.pt (T.F.); calhelha@ipb.pt (R.C.C.); carlap@ipb.pt (C.P.); iferreira@ipb.pt (I.C.F.R.F.); vcadavez@ipb.pt (V.C.); 2 The Hague University of Applied Sciences, Johanna Westerdijkplein 75, room Oval 1.01, 2521 EH The Hague, The Netherlands; rody_dijkshoorn@hotmail.com (R.D.); m.l.maloncy@hhs.nl (M.M.); 3 Institute for Biological Research “Siniša Stanković”–National Institute of Republic of Serbia, University of Belgrade, Bulevar despota Stefana 142, 11000 Belgrade, Serbia; dejanbio@ibiss.bg.ac.rs (D.S.); mris@ibiss.bg.ac.rs (M.S.)

**Keywords:** alternative flour, Algarrobo flour, nutritional quality, antioxidant, antimicrobial, cytotoxicity, breadmaking, dough rheology, texture profile analysis, alveoli size

## Abstract

Although the nutritional profile, bioactivities, and uses of mesquite pod flour from various *Prosopis* species have been studied, limited research has been conducted on *Prosopis pallida* (Humb, & Bonpl. Ex Willd.) Kunth mesquite flour. This study aimed to characterize the nutritional quality and bioactive properties of *P. pallida* pod flour and to assess its technological performance in breadmaking as a partial replacer of white wheat flour. Peruvian *P. pallida* mesquite flour was found to have an appealing nutritional profile, with high contents of dietary fiber (29.6% dw) and protein (9.5% dw), and low contents of fat (1.0% dw) and carbohydrates (57.6% dw). It is a source of palmitic (12.6%), oleic (35.5%), and linoleic acids (45.8%), α-, β-, and γ- tocopherols, and contains phenolic compounds such as apigenin glycoside derivatives with proven antioxidant capacities. Extracts of *P. pallida* flour were also found to have antimicrobial and antifungal effects and did not show hepatoxicity. When formulated as a wheat flour replacer, increasing mesquite flour levels yield composite doughs of lower stickiness and extensibility, and composite breads of lower elasticity (*p* < 0.01). However, up to a level of 10%, mesquite flour significantly increases loaf volume, reduces crumb hardness, and produces a more uniform crumb of small size alveoli (*p* < 0.01). Considering the purpose of improving the nutritional and technological quality of wheat flour bread, the addition of *P. pallida* pod flour can be highly recommended.

## 1. Introduction

The genus *Prosopis* comprises 44 species of nitrogen-fixing trees that are mainly distributed in the arid or semiarid regions of America. *Prosopis* pods are sweet fruits formed of 70–75% pericarp (epicarp, mesocarp, and endocarp) and 25–30% seeds (episperm, endosperm, and cotyledons) [1]. Whole ripe pods of *Prosopis* spp. are ground to produce flour, known as mesquite or Algarrobo flour, which is brown, sweet, and with an aroma that resembles coffee, cocoa, molasses, and hazelnut [2].

Different researchers have reported on the nutritional profile and bioactivities of mesquite flour from various *Prosopis* species. Pod flour of Mexican *P. laevigata* was found to have a good nutritional profile (10% protein, 3.6% fat, 26.7% crude fiber, and 56.8% carbohydrates) and protein digestibility (78%) [3]. Furthermore, it has been pointed out that *P. laevigata* pod flour is a good source of lysine, sulfur-containing amino acids, and total phenolic compounds, with higher radical scavenging capacity than common beans [4]. In a subsequent article [5], it was found that *P. laevigata* seeds flour maintains the healthy nutritional traits of low-fat content (4.8%), high protein (36.5%), and crude fiber (7.7%) found in legumes, whereas four amino acids (Glu, Arg, Asp, and Leu) represented more than 45% of total amino acids in their samples.

Mesquite flour from the Argentinian species (*P. alba*) is likely to be the most widely investigated one. According to Sciammaro et al. [1] and Bigne et al. [6], *P. alba* pod flour presents not only high contents of soluble sugars, dietary fiber and proteins of good quality, but is also rich in iron, calcium, vitamin C, and contains polyphenols with antioxidant activity. The flour made from *P. alba* seeds also shows high levels of proteins, minerals, fiber, and phenolic compounds, mainly flavones, with low contents of total carbohydrates and fats [7]. Other authors [8] investigated the functional properties as well as the genotoxicity of *P. alba* pod flour. They proved that this flour is a rich source of antioxidant compounds that could help prevent pathologies associated with oxidative stress. Furthermore, Cattaneo et al. [9] found that *P. alba* seed extracts enriched in polyphenolic compounds exhibited ABTS+ reducing capacity and scavenging activity of H_2_O_2_, and were able to inhibit phospholipase, lipoxygenase, and cyclooxygenase, three pro-inflammatory enzymes.

It is therefore not surprising that, due to its nutritional and functional properties, mesquite flour has been widely advocated as a health-promoting ingredient in food formulations. In breadmaking, studies have shown promising results with partial substitutions of wheat flour to produce composite bread [6,10], sweet baked products [11], and panettone-like bread [12].

Nonetheless, there are no thorough reports up to the moment regarding the bioactivity profile of mesquite flour from *Prosopis pallida*, a species native to the semiarid coastal region of Peru, despite its attractive nutritional profile [13], nor any investigation on the technological quality of bread partially formulated with this flour. Therefore, the objective of this study was twofold: (i) to characterize the nutritional value (including free sugars, fatty acids, tocopherols, and phenolic compounds), the antioxidant, anti-inflammatory, and antimicrobial activities, and the cytotoxic effects of *P. pallida* pod flour; and (ii) to evaluate the technological quality attributes of dough and bread formulated with *P. pallida* pod flour as a partial replacer of white wheat flour.

## 2. Materials and Methods

### 2.1. Chemical Composition of Mesquite Pod Flour

#### 2.1.1. Nutritional Value

*P. pallida* flour derived from mature pod mesocarp was purchased in a commercial establishment (Brand Nutrimix, Lima, Peru), and maintained in a polyethylene container at room temperature (~20 °C) throughout the analysis. The proximate composition (moisture, proteins, fat, carbohydrates, and ash) of mesquite flour was determined using AOAC procedures [14]. The protein content (N × 6.25) of the samples was estimated by the macro-Kjeldahl method; the fat content was determined by extraction using a Soxhlet apparatus, and the ash content was determined by incineration at 550 °C for at least 8 h. Total carbohydrates were calculated by difference. The energy was calculated according to the following equation: Energy (kcal) = 4 × (g protein+g carbohydrate) + 9 × (g fat). Analyses were carried out in triplicate.

#### 2.1.2. Free Sugars

HPLC system coupled to a refraction index detector (RI detector Knauer Smartline 2300) was used to determine the composition of mesquite flour sugar, as previously described in Barros et al. [15]. Sugars were identified by comparing the relative retention times. The equipment consisted of an integrated system with a pump (Knauer, Smartline system 1000, Berlin, Germany), degasser system (Smartline manager 5000), autosampler (AS-2057 Jasco), and an RI detector (Knauer Smartline 2300). Data were analyzed using Clarity 2.4 Software (DataApex, Prague, Czech Republic). The chromatographic separation was achieved with a Eurospher 100-5 NH2 column (5 µm, 250 mm × 4.6 mm i.d., Knauer) operating at 35 °C (7971 R Grace oven). The mobile phase was acetonitrile/deionized water, 70:30 (v/v) at a flow rate of 1 mL/min. Quantification was achieved using the internal standard (IS, melezitose, Sigma-Aldrich, St. Louis, MO, USA) method and by calibration curves from commercial standards. Analyses were carried out in triplicate, and results were expressed in g per 100 g of dry weight.

#### 2.1.3. Fatty Acids

Fatty acids were determined as described in Barros et al. [15], using a GC-FID (DANI model GC 1000 instrument, Contone, Switzerland.) with a Macherey–Nagel (Düren, Germany) column (50% cyanopropyl-methyl-50% phenylmethyl polysiloxane, 30 m × 0.32 mm i.d. × 0.25 µm df). The program used was as follows: the initial temperature of the column was 50 °C, held for 2 min, then a 30 °C/min ramp to 125 °C, 5 °C/min ramp to 160 °C, 20 °C/ min ramp to 180 °C, 3 °C/min ramp to 200 °C, 20 °C/min ramp to 220 °C and held for 15 min. The carrier gas (hydrogen) flow-rate was 4.0 mL/min, measured at 50 °C. Split injection (1:40) was carried out at 250 °C. Fatty acid identification was made by comparing the relative retention times from samples with standards (fatty acid methyl esters (FAME) reference standard mixture 37, Sigma-Aldrich, St. Louis, MO, USA). The fatty acids quantified were: caproic acid (C6:0); caprylic acid (C8:0); undecanoic acid (C11:0); dodecanoic acid (C12:0); myristoleic acid (C14:0); pentadecanoic acid (C15:0); palmitic acid (C16:0); palmitoleic acid (C16:1); heptadecanoic acid (C17:0); stearic acid (C18:0); oleic acid (C18:1n9); linoleic acid (C18:2n6); α-linolenic acid (C18:3n3); arachidic acid (C20:0); eicosenoic acid (C20:1); eicosadienoic acid (C20:2). Analyses were carried out in triplicate, and results were expressed in the relative percentage of each fatty acid.

#### 2.1.4. Tocopherols

The analysis was performed by HPLC (equipment described above), and a fluorescence detector (FP-2020; Jasco, Easton, MD, USA) programmed for excitation at 290 nm and emission at 330 nm. The chromatographic separation was achieved with a Polyamide II normal-phase column (5 µm, 250 mm × 4.6 mm i.d., YMC Waters), operating at 35 °C. The mobile phase used was a mixture of n-hexane and ethyl acetate (70:30, v/v) at a flow rate of 1 mL/min. Chromatographic comparisons identified the compounds with authentic standards. Quantification was based on the fluorescence signal response of each standard, using the IS (Tocol, Matreya, Pleasant Gap, PA, USA) method and by using calibration curves obtained from commercial standards of each compound [15]. Analyses were carried out in triplicate, and results were expressed in µg per 100 g of dry weight.

### 2.2. Phenolic Compounds Analysis of Mesquite Flour Extract

The lyophilized extract obtained from mesquite flour (1 g) was extracted with 30 mL of ethanol:water 80:20 (*v*/*v*) at room temperature, under agitation (150 rpm) for 1h. The extract obtained was filtered through a Whatman nº 4 paper. This procedure was repeated twice, and then the extracts were evaporated at 35 °C (rotary evaporator Büchi R-210) to remove ethanol. The aqueous phase of the extraction was purified to remove sugars and polar substances, increasing the concentration of the phenolic compounds. For this, a C-18 SepPak^®^ Vac 3 cc cartridge (Phenomenex) was used, previously activated with methanol followed by water. The extract was taken to dryness by rotary evaporator, re-dissolved in 1 mL of ethanol:water 80:20 (*v*/*v*), and filtered through a 0.22-µm disposable LC filter disk for HPLC analysis [16]. The chromatographic data were acquired from Dionex Ultimate 3000 UPLC (Thermo Scientific, San Jose, CA, USA), as previously described in Bessada et al. [17]. Chromatographic separation was achieved with a Waters Spherisorb S3 ODS-2 C18 (3 μm, 4.6 × 150 mm, Waters, Milford, MA, USA) column thermostated at 35 °C. The solvents used were: (A) 0.1% formic acid in water, (B) acetonitrile. The elution gradient established was isocratic 15% B (5 min), 15–20% B (5 min), 20–25% B (10 min), 25–35% B (10 min), 35–50% B (10 min), and re-equilibration of the column, using a flow rate of 0.5 mL/min and an injection volume of 10 μL. Double online detection was carried out in the DAD using 280, 330, and 370 nm as preferred wavelengths. MS detection was performed in negative mode, using a Linear Ion Trap LTQ XL mass spectrometer (Thermo Finnigan, San Jose, CA, USA) equipped with an ESI source. Nitrogen served as the sheath gas (50 psi); the system was operated with a spray voltage of 5 kV, a source temperature of 325 °C, and a capillary voltage of −20 V. The tube lens offset was kept at a voltage of −66 V. The full scan covered the mass range from *m*/*z* 100 to 1500. Data acquisition was carried out with Xcalibur^®^ data system (Thermo Finnigan, San Jose, CA, USA). The characterization of the phenolic compounds present in the samples was made according to their UV-Vis spectra, mass, and retention time, comparing with the standards, when available. When this was not possible, data from literature was used to provisionally identify the compounds. Quantitative analysis was performed for each identified compound using the calibration curves for each available phenolic standard, as well as the most appropriate UV-Vis signal for each identified compound. The phenolic compounds detected that did not have a commercial standard, were quantified using the most similar commercial standard calibration curve available. The analyses were carried out in triplicate, and results were expressed as mean values and standard deviations (SD), in mg/g of lyophilized extract.

### 2.3. Bioactivities of Mesquite Flour Extract

#### 2.3.1. Antimicrobial Activity Assays

The antibacterial activity of the previously described hydroethanolic extract of mesquite flour was evaluated according to Soković et al. [18], using Gram-positive bacteria (*Listeria monocytogenes*: NCTC 7973 and *Staphylococcus aureus*: ATCC 6538) as well as Gram-negative bacteria (*Escherichia coli*: ATCC 25922, *Salmonella* Typhimurium: ATCC 13311, and *Enterobacter cloacae*: human isolate). The minimum inhibitory (MIC) and minimum bactericidal (MBC) concentrations were determined, and streptomycin and ampicillin were used as positive controls. In contrast, the antifungal activity of the hydroethanolic extract of mesquite flour was evaluated following the protocol described in Soković and van Griensven [19], using *Aspergillus niger* (ATCC 6275), *Aspergillus fumigatus* (ATCC 1022), *Penicillium ochrochloron* (ATCC 9112), *Penicillium funiculosum* (ATCC 36839), *Penicillium verrucosum* var. *cyclopium* (food isolate), and *Aspergillus ochraceus* (ATCC 12066). The MIC and MFC (minimum fungicidal concentration) were determined. Ketoconazole and bifonazole were used as positive controls. All measurements were obtained in triplicate. The microorganisms are deposited at the Institute for Biological Research “Siniša Stanković”—National Institute of the Republic of Serbia, University of Belgrade.

#### 2.3.2. Antioxidant Activity Assays

The antioxidant activity of the hydroethanolic extract obtained from mesquite flour was evaluated using two cell-based methodologies. The lipid peroxidation inhibition in porcine (*Sus scrofa*) brain homogenates was evaluated by the decrease in thiobarbituric acid reactive substances (TBARS) following the protocol described in Pinela et al. [20]. The analysis was carried out in triplicate, and results were expressed in EC_50_ values (sample concentration providing 50% of antioxidant activity, µg/mL). The anti-hemolytic activity of the extracts was evaluated by the oxidative hemolysis inhibition assay (OxHLIA), as described in Lockowandt et al. [21]. This analysis was replicated three times, and results were expressed as the inhibitory concentration (IC_50_ value, µg/mL) able to promote a Δt hemolysis delay of 60 and 120 min. Trolox (Sigma Chemical Co.) was used as a positive control for both assays.

#### 2.3.3. Cytotoxic Activity Assays

The cytotoxicity was determined in the mesquite flour hydroethanolic extract using four human tumor cell lines, HeLa (cervical carcinoma), HepG2 (hepatocellular carcinoma), MCF-7 (breast adenocarcinoma), and NCI-H460 (non-small cell lung cancer), and a non-tumor primary culture PLP2 (porcine liver). Dulbecco’s modified Eagle’s medium (DMEM) supplemented with FBS (10%), penicillin (100 U/mL), and streptomycin (100 μg/mL) were used [22]. The cell growth inhibition was measured using the sulforhodamine B (SRB) assay. Analyses were carried out in triplicate, and the results were expressed as GI_50_ values (sample concentration that inhibited 50% of the net cell growth, in μg/mL), and ellipticin was used as a positive control.

### 2.4. Technological Performance of Mesquite Flour in Breadmaking

Wheat white flours of type 55 (11.0% protein, 0.51% ashes) and type 65 (12.5% protein, 0.63% ashes) were obtained from Loreto Mills (Bragança, Portugal). Refined and non-iodinated salt and dried yeast were purchased from a local supermarket (Bragança, Portugal). Distilled water kept at 4 °C was used to prepare the bread. A full two-factor design was employed, varying the replacement of wheat flour of either type 55 or 65 with mesquite flour (0, 5, 10, and 15% w/w), which produced a total of 8 treatments repeated twice.

#### 2.4.1. Breadmaking Process

Bread loaves for all of the treatments were prepared following the same method. For each batch, a dough of ~1600 g was prepared. Each batch was prepared by mixing the dry ingredients (wheat flour 55 or 65, mesquite flour, yeast at 5% total flour weight and 1.5% salt) for 2 min at speed 2 using a batter blade in a professional food processor (SilverCrest SKMP-1200, Germany). Water (59.1%) was added and mixed for 2 min at speed 1 followed by 7 min at speed 2 using a dough hook. A dough portion of ~150 g was spared for the analysis of rheological properties. The rest was shaped like a cylinder and placed in a floured tray to proof as bulk for 60 min in a climate chamber at 37 °C and 85% RH (Climacell 222, Boston, MA, USA). Then, the dough was carefully portioned into six 230 ± 10-g balls and placed into oiled and floured tins. The tins had a base dimension of 12 cm × 8 cm. These were then placed in the same climate chamber (37 °C and 85% RH) and allowed to proof for an additional 15 min. Subsequently, the tins and trays were placed in a preheated convection oven (Princess, 2000W, The Netherlands) at 230 °C and baked for 40 ± 0.5 min. After baking, the bread loaves were removed from the tins and allowed to cool at room temperature (20–25 °C). Analyses were carried out 24 h after baking.

#### 2.4.2. Rheological Analyses of Dough

Approximately 5 g dough was weighed in an SMS/Chen-Hoseney stickiness cell (A/DSC) screwed to an SMS/Chen-Hoseney stickiness rig [23]. Approximately 1-mm dough was extruded through the rig and analyzed by the texture analyzer TA-XT plus, implemented with Exponent software version 6.1.11.0 (Stable Micro Systems, UK). A 25-mm perspex cylinder probe (P/25P) was used for the analysis. The parameters during the test were set at: pre-test speed 0.5 mm/s, test speed 0.5 mm/s, and post-test speed 10 mm/s, trigger force was set at 5 g, applied force 40 g, contact time 0.1 s, and return distance 4 mm. The obtained parameters were: dough stickiness, work of adhesion (g.s), and dough cohesive-strength (mm).

Extensibility analysis was performed using a Kieffer Dough & Gluten Extensibility Rig (Stable Micro Systems, UK). The dough was flattened to a sheet of 5 mm (±0.5 mm) and cut into a rectangle of 15.0 × 5.1 cm, and placed on top of a lubricated (sunflower seed oil) Teflon base plate. A lubricated rigged Teflon top plate was adjusted on top of the dough and pressed using a clamp. The clamp was screwed tightly so that the rigs cut the dough sheet into strips. The plates were held in place for 10 min to relieve any stresses present in the dough caused by squeezing. The top plate was removed and ~5 cm long dough strips with a cross-section that resembles a trapezium were produced. The first 3 strips of each side were discarded. Subsequently, a dough strip was carefully placed on a U-shaped plate and clamped on the base plate. A hook with rod 1.20 mm was used for the analysis of the dough [24]. The test was set at a lifting speed of 3.30 mm/s and a distance of 75 mm, and the parameters obtained were extensibility (mm) and resistance to extension (g). Each of the dough analyses was repeated 10 times.

#### 2.4.3. Physicochemical Analyses of Bread

The volume of each loaf (ml) was determined with the use of a modified version of the standard rapeseed displacement method 10-05 [25], using quinoa seeds instead of rapeseeds. Volume was determined in triplicate and for four different loaves per treatment. The specific volume of a loaf (ml/g) was obtained by dividing the volume by the loaf weight after 24 h baking. Baking loss (%) was calculated as [initial loaf weight before baking—loaf weight after 24 h baking]/[initial loaf weight before baking] × 100 for four loaves per treatment. Loaves were sliced into 1.0 cm (±0.05 cm) thick slices using an electric slicer (Bosch MAS4000W, Damme, Germany). Measurements of the water activity (aw) of the bread crumb were obtained at 20 °C with the use of an Aqualab (4TE Decagon, Munich, Germany). Measurements were taken from three central slices of each of two loaves per treatment.

#### 2.4.4. Bread Crumb Texture Analysis

On the day of analysis, bread slices of 15-mm thick were obtained from two loaves per treatment. The first and last two slices of each loaf were discarded. A 30-kg load cell was used for calibration of the texture analyzer. A 50-mm diameter dough cutter was used to cut off a crumb cylinder from the center of four slices per bread loaf (amounting to 8 TPA repetitions per treatment). A 40 mm-diameter probe was fitted to the texture analyzer. The parameters for the test were set at: pre-test speed 1 mm/s, test speed 2 mm/s, and post-test speed 2 mm/s, trigger force 5 g, 50% sample deformation (strain), and double compression (with a 30 s interval between cycles). The TPA parameters obtained were: hardness (g), springiness (dimensionless), cohesiveness (dimensionless), chewiness (g), and resilience (dimensionless).

#### 2.4.5. Image Analysis of Bread Crumb Features

For crumb image analysis, two loaves were used per formulation. Loaves were sliced, and each slice—except the extreme ones—was scanned on both sides (Pixma MG-2550, Canon Portugal, Porto, Portugal) using IJ Scan Utility software (version 2.0,12, Canon, Tokyo, Japan). The parameters were set at the grey level at −10% brightness, +15% contrast, and 350 dpi resolution. ImageJ software (version 1.518, Wayne Rasband, National Institutes of Health, Bethesda, MD, USA) was employed to crop the center of the image into a 3.8 cm × 3.8 cm field-of-view sub-image (spatial resolution 1 cm = 138 pixels). Each image was saved without any image compression in TIF format. Each formulation, therefore, produced 16 images (2 loaves × 4 slices × 2 sides). A binary segmentation procedure based on the k-means clustering algorithm, proposed by [26] was used to obtain several grain crumb features, using Matlab software (version R2015a, The Mathworks, USA). The parameters obtained were mean cell area (mm^2^), mean cell density (nº cells/mm^2^), cell size uniformity (dimensionless, which is calculated as the ratio between the number of cells ≤5 mm^2^ and number of cells >5 mm^2^), the void fraction (dimensionless, which is calculated as the proportion of the occupied two-dimensional space by cells), mean cell compactness (dimensionless, defined as the ratio of the area of the cell to the area of a circle having the same perimeter), and mean cell aspect ratio (dimensionless, defined as the ratio between the major axis and the minor axis of the cell).

### 2.5. Statistical Analysis

Results of the nutritional and bioactive properties are presented as means and standard deviations. The separate effects and interaction of mesquite flour substitution level (0%, 5%, 10%, and 15%) and wheat flour type (T65 and T55) on baking loss, loaf specific volume, a_w_ and dough rheology properties were assessed by analysis of variance, performed through a simple linear model. Instead, the statistical analyses of the bread crumb TPA properties and crumb image analysis features were carried out by a linear mixed model because the measurements taken from the same loaf had to be grouped. For all technological quality properties, Tukey’s Honest Significant Difference test was performed when effects were significant (α = 0.5). To reduce the small sample bias, the Kenward–Roger correction was applied [27]. Models were fitted in R studio 1.0.136 implemented in R version 3.3.2.

## 3. Results and Discussion

### 3.1. Nutritional Value of Prosopis pallida Pod Flour

The results concerning the nutritional value, free sugars, and fatty acids composition are presented in Table 1. Carbohydrates were the major constituents (57.6 ± 0.1 g/100 g dw) of *P. pallida* pod flour, followed by proteins (9.5 ± 0.1 g/100 g dw). Fat contents were low (1.0 ± 0.1 g/100 g dw) and ash content was 2.3 ± 0.1 g/100 g dw. The energetic contribution at 388.3 ± 0.1 kcal/100 g dw is in agreement with previous research conducted on *P. pallida* flour of the same geographical origin (362 kcal/100 g dw in Felker et al. [2]). Nevertheless, the proximate composition presented in such an early study is slightly different, with a lower content of proteins (8.1 g/100 g dw), and higher contents of carbohydrates (82.6 g/100 g dw) and ashes (3.6 g/100 g dw).

Total sugars of mesquite flour were calculated at 18.3 ± 0.2 g/100 g, consisting of 17.5 ± 0.2 g/100 g of sucrose, 0.59 ± 0.02 g/100 g of fructose, and 0.131 ± 0.004 g/100 g of glucose; all of which were contents much lower than those reported for P. alba pod flour by Felker et al. [13] (38.2, 9.1, and 2.6 g/100 g dw, respectively). The total dietary fiber of Peruvian mesquite flour was at 29.6 ± 0.2 g/100 g dw, split into 26.3 ± 0.4 g/100 g dw for insoluble fiber and 3.3 ± 0.2 g/100 g dw for soluble fiber. These results are comparable with those encountered by Felker et al. [2] of 32.2, 30.6, and 1.62 g/100 g dw, respectively, for *P. pallida* pod flour from the same geographical region.

Regarding the fatty acid profile (Table 1), palmitic acid (C16:0; 12.6%), oleic acid (C18:1n9c; 35.5%), and linoleic acid (C18:2n6c; 45.8%) were the main constituents, which was in agreement with the main fatty acids in Mexican *P. laevigata* flour [28], although in different proportions (18.8, 23.6, and 43.6%, respectively). Polyunsaturated fatty acids (PUFA; 47.7%) of *P. pallida* flour predominated over monounsaturated fatty acids (MUFA; 36.0%), as was encountered for *P. laevigata* flour, even though in different proportions (52.3 and 23.6%, respectively, in Cruz-Gracida et al. [28]). Concerning the tocopherols, α, β, and γ isoforms were found, being γ-tocopherol the most predominant one with 1.81 ± 0.01 g/100 g dw.

### 3.2. Phenolic Compounds in Prosopis pallida Pod Flour

Regarding phenolic compounds (Table 2), seven compounds were identified and quantified. Isorhamnetin-O-rutinoside (peak 6) was positively identified according to its retention time, mass and UVvis characteristics by comparison with commercial standards. The compounds 1 to 5 were identified as C-glycosyl flavones as previously described by several authors [4,9,29,30] in *Prosopis* species. Peaks 2 to 5 ([M-H]^−^ at *m*/*z* 563) all presented the same pseudomolecular ion, with similar fragmentation patterns, which is characteristic of di-C glycosides. The produced ions at *m*/*z* 545 [M-H-18]^−^, *m*/*z* 503 [M-H-60]^−^ and a base peak at *m*/*z* 473 [M-H-120]^−^, suggested a hexosyl and pentosyl as the sugar moieties. The fragment ions at *m*/*z* 353 [aglycone + 83]^−^ and *m*/*z* 383 [aglycone + 113]^−^ suggest that the aglycone is apigenin; thus, these compounds were identified as apigenin-C-hexoside-C-pentoside isomers. This identification is also in agreement with that reported in Prosopis pods and syrups by Pérez et al. [31] and Quispe et al. [32], in which schaftoside (apigenin-8-C-arabinoside-6-C-glucoside) and isoschaftoside (apigenin-6-C-arabinoside-8-C- glucoside) were identified. Peak 1 ([M-H]^−^ at *m*/*z* 593) was identified as apigenin-6,8-di-C-glucoside (vicenin), due to the observation of the ions at *m*/*z* 473 and 353 (loss of two hexosyl moieties), which points out to the presence of a di-C-hexosyl derivative. In phenolic-enriched cotyledon flour extracts from Argentinian *P. alba*, Cattaneo et al. [9] also identified that the main C-glycosyl flavones were schaftoside, isoschaftoside, vicenin II, vitexin, and isovitexin. Other works have also reported the presence of C-glycosides of apigenin in the pod flour of Argentinian *P. nigra* [33], and the seed flour of Mexican *P. laevigata* [5].

### 3.3. Bioactivity Profile of Prosopis pallida Pod Flour

The results of bioactivities are presented in Table 3. Two methods were used for antioxidant activity: the lipid peroxidation inhibition (TBARS) and the oxidative hemolysis inhibition (OxHLIA). In both assays, *P. pallida* pod flour exhibited antioxidant capacity. The same conclusion was arrived at earlier by Silva Pinto et al. [34] who, by means of another in vitro assay, determining values of DPPH scavenging activity of 85% for aqueous extract and 89% for 12% ethanolic extract of *P. pallida* pod flour. The antioxidant activity of Peruvian *P. pallida* flour extracts could be attributed to the C-glycosyl flavonoids, which possess proved antioxidant capacity [35]. In the case of *P. alba*, sugar-free polyphenolic extracts of pods also demonstrated high antioxidant activity [8] whereas organic extracts enriched from polyphenolic compounds obtained from cotyledons flour exhibited high reducing capacity with SC_50_ values of 7.1 μg GAE/mL [9].

The mesquite extract was also studied regarding their antimicrobial properties. To this end, three Gram-negative (*E. coli*, *E. cloacae*, and *S. typhimurium*) and two Gram-positive (*L. monocytogenes* and *S. aureus*) bacteria were selected. The results obtained revealed that the *P. pallida* mesquite extract was effective against all the tested organisms. Furthermore, those results suggest a selective action against Gram-negative strains. The achieved MIC values for the Gram-positive bacteria range between 0.60 and 0.40 mg/mL in *L. monocytogenes* and *S. aureus*, respectively; whereas the results for the Gram-negative bacteria are considerably lower (0.20 mg/mL). The lowest MIC value (highest antibacterial activity) was observed in *E. coli* and *S.* Typhimurium (MIC = 0.20 mg/mL) when compared to the positive control (ampicilin).

These findings allied to the fact that none of the extracts showed MBC up to 1.20 mg/mL, make mesquite extracts good candidates for applications in food and food supplement formulation, aiming to increase shelf-life by retarding the proliferation of Gram-positive bacteria. In this regard, Arya et al. [36] used a leaf extract obtained from *P. juliflora* to provide anti-biofilm activity. Other authors revealed promising antimicrobial effects in several *Prosopis* species [37,38,39].

Concerning the anti-fungal activity, mesquite flour extract was effective against *A. ochraceus*, *P. ochrochloron*, *P. funiculosum*, and *P.v. cyclopium*; however, only *P.v. cyclopium* presented lower MIC values in comparison to the positive control, proving favorable results, which have also been previously noticed by other authors [40,41]. By contrast, it did not reveal effectiveness against *A. fumigatus* and *A. niger*.

Regarding cytotoxic effects, the pod flour of *P. pallida* shown to be selective for NCI-H460 cell line (242 ± 4 µg/mL), as no activity was observed against the other cell lines (MCF-7, HeLa, and HepG2) studied. Nevertheless, it also did not show hepatotoxicity in the primary cell culture (non-tumor cells; PLP2) applied in this study, revealing no toxicity up to the maximum concentration studied (GI_50_ at >400 µg/mL). The results about the cytotoxic activity of *P. pallida* or *P. alba* pod flour extracts are scarce in literature; however, some authors showed that *P. juliflora* leave extracts caused a significant reduction in cell viability in various human cancer cell lines [42,43,44].

### 3.4. Technological Quality of Prosopis pallida Pod Flour in Breadmaking

**Figure 1:**
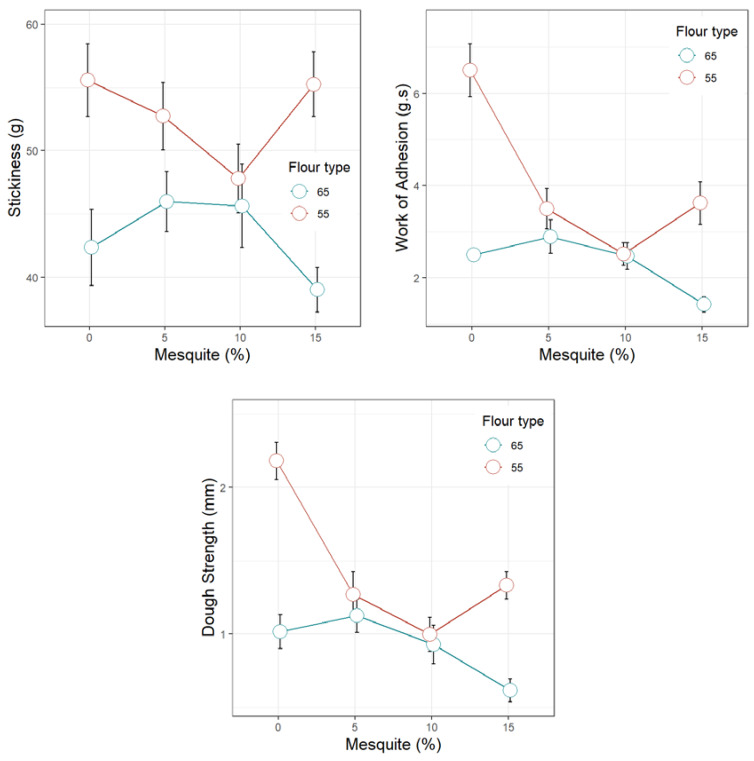
Stickiness properties of dough made of wheat flour type 65 or 55 partially replaced with mesquite pod flour.

**Figure 2:**
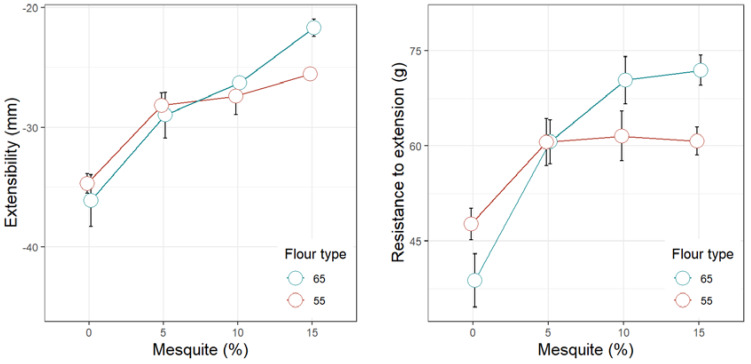
Extensibility properties of dough made of wheat flour type 65 or 55 partially replaced with mesquite pod flour.

**Figure 3:**
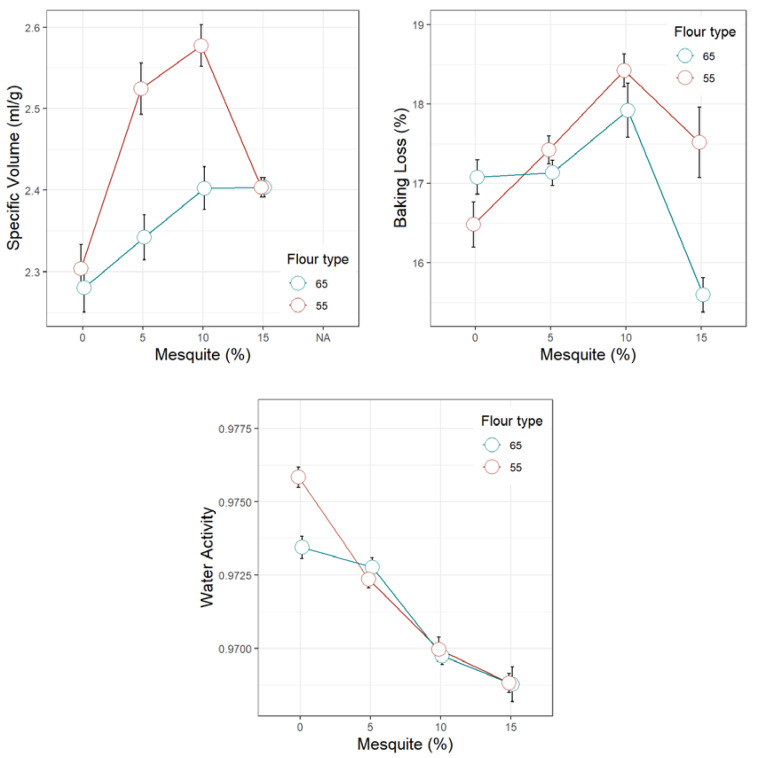
Loaf gravimetric properties and crumb water activity of bread made of wheat flour type 65 or 55 partially replaced with mesquite pod flour.

**Figure 4:**
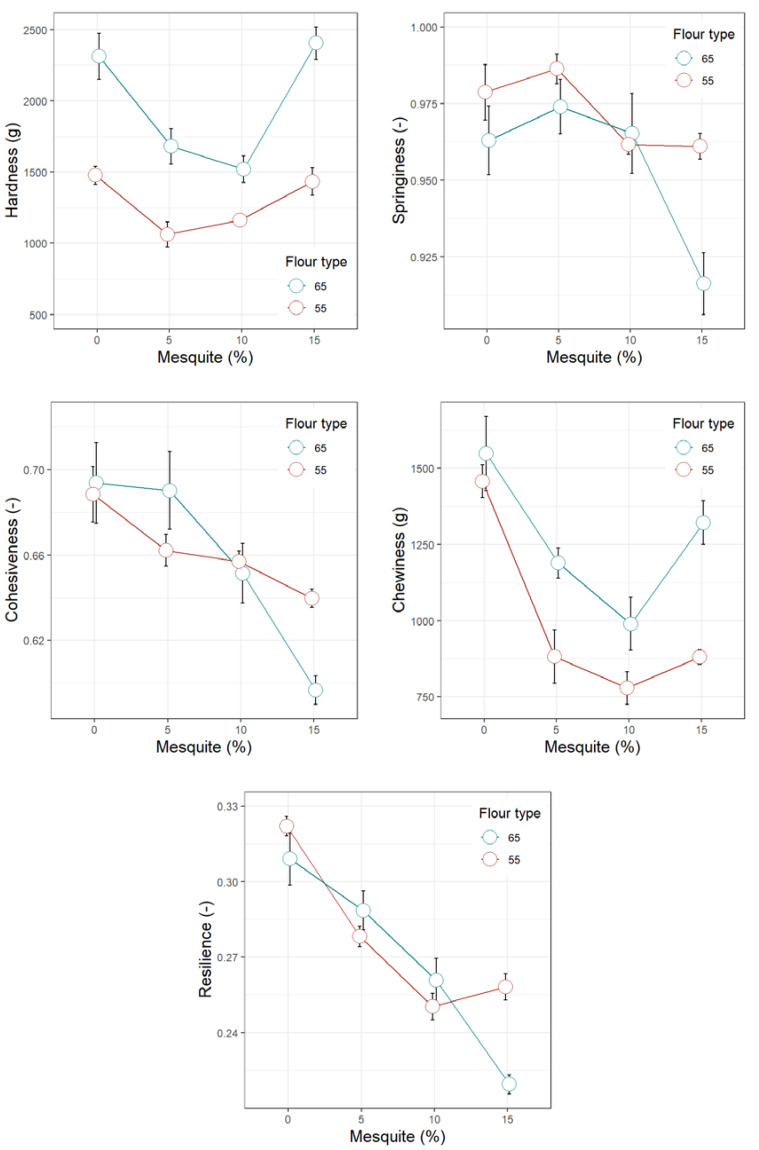
Crumb texture profile analysis features of bread made of wheat flour type 65 or 55 partially replaced with mesquite pod flour.

**Figure 5:**
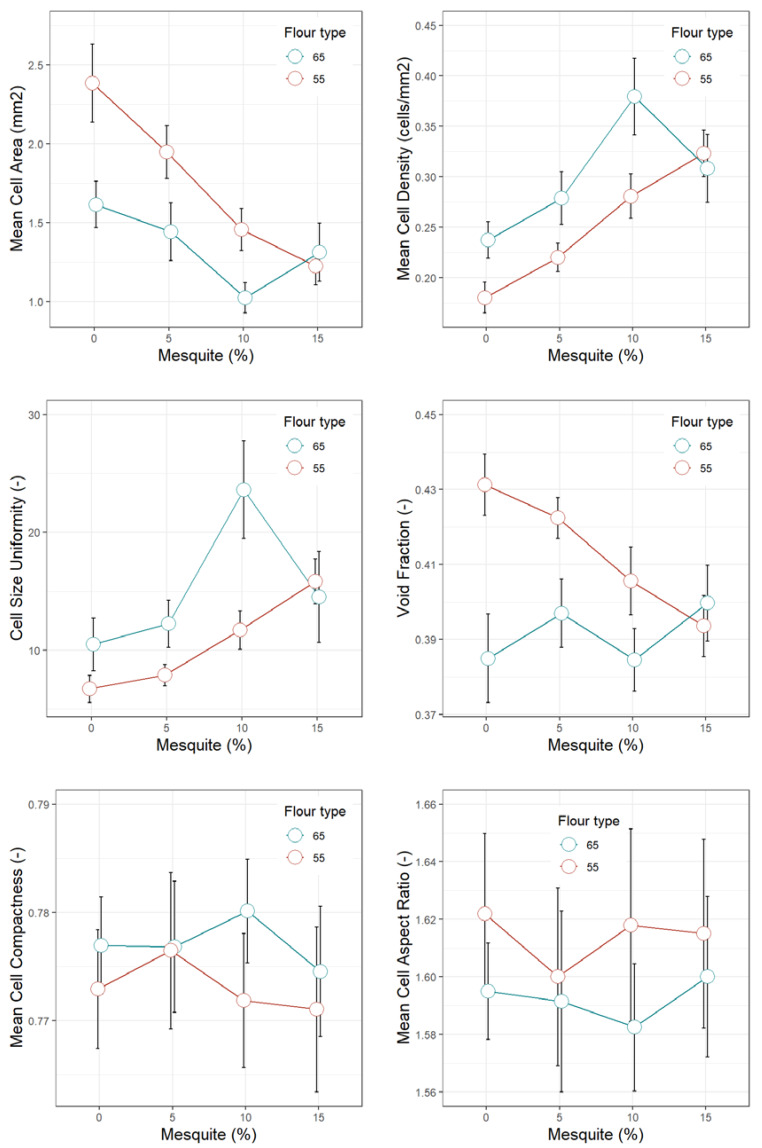
Image analysis crumb features of bread made of wheat flour type 65 or 55 partially replaced with mesquite pod flour.

Figure 1, Figure 2, Figure 3, Figure 4 and Figure 5 display the trends of technological quality properties of composite bread, as affected by replacing wheat flour type 65 or 55 with different levels of mesquite flour (0%, 5%, 10%, 15%). Table A1, Table A2, Table A3 and Table A4 given in Appendix A, compile the full results of the analysis of variance for the effects of white wheat flour type, mesquite flour substitution level, and their interaction, as well as the mean and standard errors of the technological quality properties for the main effects.

#### 3.4.1. Rheology of Wheat Flour Dough Partially Replaced with Mesquite Flour

Mesquite flour affected the properties of dough stickiness (Figure 1) and dough extensibility (Figure 2), yet to a different extent depending on the wheat flour type. In general, when added to wheat flour T55, mesquite flour produced composite doughs with greater stickiness, work of adhesion, and strength than when mixed with wheat flour T65. As the level of substitution of wheat flour T65 with mesquite flour increases, stickiness, work of adhesion, and strength steadily fall; this fall is more pronounced when wheat flour T55 is used (except for the 15% mesquite level). This behavior is likely to be a result of a weaker gluten network formed during mixing due to the greater amounts of water immobilized by the fiber and galactomannans conferred by the mesquite flour. Since wheat flour T55 has lower gluten content than T65, the reduction in the stickiness properties is more marked when mesquite flour is added to the former and becomes evident by the abrupt drop in dough strength and work of adhesion between the control (0% mesquite flour) and 5% mesquite flour (Figure 1). Correa et al. [11] also observed that *P. alba* pod flour in mixture with wheat flour produced softer and less consistent doughs. The weakness of the dough network can be attributed to the dilution of the gluten and/or the presence of certain mesquite flour components, such as the galactomannans, that could modify the interaction among gluten proteins.

Increasing levels of mesquite flour decreased the extensibility of the dough (*p* < 0.0001) and increased the dough resistance to extension (*p* < 0.0001), whereas composite doughs of wheat flour T65 presented lower extensibility and higher resistance to extension than those of wheat flour T55 (*p* < 0.0001). Nonetheless, the effect of mesquite flour on the extensibility properties of the composite dough was less marked when combined with wheat flour T55, as some stabilization in both extensibility and resistance to extension was achieved between the substitution levels of 5% and 15% (Figure 2; Table A1). In other words, mesquite flour produces a greater loss in dough extensibility when mixed with wheat flour T65. In addition to the factors early discussed as causing a deficient gluten network development—i.e., gluten dilution and galactomannans—the additional fiber supplemented by the increasing levels of mesquite flour can also interact with the gluten proteins affecting the extensibility of the composite dough, as pointed out by Bigne et al. [45].

#### 3.4.2. Physicochemical Properties of Wheat-Mesquite Flour Bread

Both gravimetric properties of a bread loaf—baking loss and specific volume—increased for increasing mesquite flour proportions until 10%, reaching in mixture with wheat flour T55, in general, higher values than in mixture with wheat flour T65 (Figure 3). Thus, the formulation with the highest specific volume belonged to 10% substitution of wheat flour T55 with mesquite flour, which represented ~12% gain in volume with respect to the control. A breakpoint was observed beyond the substitution level of 10%. At the highest tested proportion of 15% mesquite flour, the specific volume of the formulation based on flour T65 did not further increase, while that of the formulation with flour T55 dramatically dropped although it remained higher than the control (Figure 3). The same breakpoint was observed for baking loss beyond the 10% substitution level of mesquite flour. The beneficial effect of mesquite flour on loaf volume, found in the present study, is in disagreement with Bigne et al. [12] and Correa et al. [11] who estimated that replacing wheat flour with 15% or 30% *Prosopis alba* pod flour produced a loss in loaf volume of ~13% or 15%, respectively.

These findings may not be comparable with the present study since the mesquite flour used was obtained from a different species (*P. pallida*) which is known to have a very distinct composition, with higher contents of protein (8.11% db), crude fibre (3.40% db), and insoluble dietary fibre (306 g/kg db), and lower contents of fat (0.77% db) and total sugars (485 g/kg db) than those of *P. alba* pod flour (7.17% db, 2.40%, 200 g/kg db, 2.17% and 591 g/kg db, respectively) [2,13].

Loaf volume is a parameter that reflects both gluten network quality and matrix capability to retain the gases formed during fermentation. The fact of gluten content being diluted by the addition of mesquite flour is counteracted by the action of the galactomannans of high molecular weight (~900,000 to 1,000,000 Da) present in Peruvian *P. pallida* seeds [46], which enable the matrix to retain the gas better. Furthermore, Sciammaro et al. [1] stated that Prosopis gums also contain proteins of globular nature, which are capable of absorbing water and form a gel-like structure. The capacity of the galactomannans and globular proteins to entrap large amounts of water also explains why the bread crumb aw significantly decreased with higher levels of mesquite flour (*p* < 0.0001; Table A2).

#### 3.4.3. Texture Profile of Wheat-Mesquite Flour Bread Crumb

All crumb texture parameters were affected by the addition of mesquite flour. Regardless of the wheat flour type, replacing them with 5% or 10% mesquite flour produced softer crumbs than the control (0%), while at a substitution level of 15%, the crumb hardness increased to the same level of the control (Figure 3). This was an expected behaviour since the loaf volume—a property inversely correlated with hardness—also increased up to a substitution level of 10% mesquite flour. The softest bread crumb was then produced by the formulation that replaced 5% of wheat flour T55 by mesquite flour. With increasing mesquite flour proportions, crumb chewiness followed the same trend as hardness, since these properties are related. Nonetheless, the crumb chewiness of all composite breads was significantly lower than the controls (0% mesquite flour) (Figure 3).

Crumb cohesiveness was not significantly affected (i.e., not different from the control) only when 5% mesquite flour replaced wheat flour T65 (Table A3). All of the other treatments added with mesquite flour significantly affected crumb cohesiveness (*p* < 0.0001), which is a negative effect since less cohesive crumbs are more susceptible to disintegration. In mixture with wheat flour T65, mesquite flour added up to a proportion of 10% did not significantly change crumb springiness, although at a substitution level of 15% the loss in springiness was highly noticeable (Figure 4). In mixture with wheat flour T55, the reduction in springiness was slight but occurred from 10% mesquite flour proportion onwards. Resilience, related to the crumb’s ability to recover after compression, progressively decreased (*p* < 0.0001) as mesquite flour proportion increased, regardless of the wheat flour type; which suggests that the addition of mesquite flour causes loss in crumb elasticity.

#### 3.4.4. Image Analysis Crumb Features of Wheat-Mesquite Flour Bread

Higher proportions of mesquite flour produced loaves with an increasingly closer crumb structure, as suggested by the lower mean cell area (*p* < 0.0001) and the higher mean cell density (*p* < 0.0001) (Figure 5, Table A4). Mesquite flour formulations produced crumbs with a higher number of small size alveoli than the control; although in mixture with wheat flour T65, alveoli were smaller and in a higher number than in mixture with wheat flour T55. As a consequence, mean cell size uniformity also tended to increase with higher proportions of mesquite flour from a mean value of 8.60 (0% mesquite) until 17.70 (10% mesquite). In mixture with wheat flour T65, mesquite flour at 15% produced crumbs of larger cell size and therefore lower mean cell size uniformity than in mixture with flour T55, which may hint that at this high level of substitution, small alveoli no longer hold in the matrix and coalesce.

The void fraction was not affected by the mesquite flour addition in the formulations with wheat flour T65. However, when replacing wheat flour T55, the addition of higher proportions of mesquite flour produced bread crumbs of progressively lower void fraction values (Figure 5).

Similarly, Bigne et al. [45] reported higher cell density, and lower cell size area and void fraction in crumbs produced with increasing amounts of *P. alba* pod flour. They also observed that the addition of *P. alba* pod flour significantly affected alveoli shape, producing cells of lower circularity or compactness. However, in our study, alveoli shape was not altered by mesquite flour either in mixture with T55 or in mixture with T65, as can be appreciated from the parameters mean cell compactness (*p* = 0.156) measuring circularity and mean cell aspect ratio (*p* = 0.383) measuring elongation (Figure 5; Table A4). Overall, in terms of cell size distribution, mesquite flour breads formulated with flour T55 yielded a better porosity than those of flour T65.

Many studies have reported that replacing even a low percentage of white wheat flour with flours of legumes such as chickpea, soy and mesquite (other than *P. pallida*) affects the rheological properties of the composite dough causing lower extensibility and tenacity, and higher resistance to extension, that ultimately induce several technological properties such as low volume, less elastic/resilient, harder and shredded crumbs, increased chewiness, and compact structures of smaller size alveoli [6,10,47]. As shown in this study, most of the above technological faults did occur when replacing white wheat flour with Peruvian *P. pallida* pod flour, except for the quality properties of loaf volume, and crumb hardness and chewiness, where the addition of this flour at substitution levels of 5%–10% exerted a beneficial effect. In this range of substitution, composite breads presented higher volume and crumbs of reduced hardness and chewiness. This may be an effect of the high molecular weight galactomannans and globular proteins present in *P. pallida* pod flour, which counteracted gluten dilution by forming a gel-like structure with increased gas entrapment capacity [1,46]. Considering *P. pallida* pod flour’s ability to reduce crumb hardness as well as the activity of its extracts against some species of fungus and Gram-positive bacteria, further studies should be conducted on the potential application of *P. pallida* pod flour as a natural ingredient or additive to extend shelf life of bread.

## 4. Conclusions

Pod flour of Peruvian *P. pallida* was found to have a good nutritional profile, characterised of high contents of dietary fibre (29.6% dw) and protein (9.5% dw), and low contents of fat (1.0% dw) and carbohydrates (57.6% dw). *P. pallida* flour is a source of palmitic, oleic, and linoleic acids, and tocopherols of the α, β, and γ isoforms, and contains phenolic compounds, such as vicenin II, isoschaftoside, and schaftoside, with proven antioxidant capacity. Extracts of *P. pallida* flour were found to have antimicrobial effect with minimum inhibitory concentrations lower than 0.30 mg/mL against *E. coli*, *S. typhimurium*, *A. ochraceous*, *P. ochrochloron*, and *P. funiculosum*; no toxicity up to the maximum concentration studied (GI_50_ at >400 µg/mL). In a future study, the endurance of bioactivities after the bread making process will be assessed.

The addition of *P. pallida* pod flour affects most of the technological quality attributes of bread, probably due to the presence of globular proteins, fibre, and high-molecular-weight galactomannans that interfere with gluten network development. When formulated as a wheat flour replacer, increasing proportions of mesquite flour produce composite doughs of lower tenacity, stickiness and extensibility, and composite bread of lower cohesiveness and elasticity. However, up to a substitution level of 10%, the addition of mesquite flour significantly increases loaf volume, reduces crumb hardness, and produces a more uniform crumb aspect consisting of more alveoli of small size. Considering the purpose of improving the nutritional and technological quality of white wheat flour bread, the use of *P. pallida* pod flour is highly recommended.

## Figures and Tables

**Table 1:** Nutritional composition of *Prosopis pallida* flour derived from mature pod expressed in dry weight basis (mean ± SD).

Nutritional Value		Fatty Acids ^1^ (%)	
**Moisture (g/100 g dw)**	3.5 ± 1	C6:0	0.01 ± 0.001
Ash (g/100 g dw)	2.3 ± 0.1	C8:0	0.02 ± 0.001
Proteins (g/100 g dw)	9.5 ± 0.1	C11:0	0.04 ± 0.003
Fat (g/100 g dw)	1.0 ± 0.1	C12:0	0.02 ± 0.001
Carbohydrates (g/100 g dw)	57.6 ± 0.1	C14:0	0.07 ± 0.004
Energy (kcal/100 g dw)	388.3 ± 0.1	C15:0	0.050 ± 0.004
Dietary Fibre (g/100 g dw)	29.6 ± 0.2	C16:0	12.6 ± 0.2
Insoluble	26.3 ± 0.4	C16:1	0.181 ± 0.004
Soluble	3.3 ± 0.2	C17:0	0.16 ± 0.02
**Free Sugars (g/100 g dw)**		C18:0	2.58 ± 0.04
Fructose	0.59 ± 0.02	C18:1n9c	35.52 ± 0.07
Glucose	0.131 ± 0.004	C18:2n6c	45.8 ± 0.2
Sucrose	17.5 ± 0.2	C18:3n3	1.67 ± 0.01
Total Sugars	18.3 ± 0.2	C20:0	0.70 ± 0.02
**Tocopherols (g/100 g dw)**		C20:1	0.31 ± 0.01
α-Tocopherol	0.55 ± 0.01	C20:2	0.17 ± 0.02
δ-Tocopherol	0.156 ± 0.004	SFA	16.3 ± 0.2
γ-Tocopherol	1.81 ± 0.01	MUFA	36.01 ± 0.07
Total Tocopherols	2.52 ± 0.01	PUFA	47.7 ± 0.3

^1^ Caproic acid (C6:0); Caprylic acid (C8:0); Undecanoic acid (C11:0); Dodecanoic acid (C12:0); myristoleic acid (C14:0); Pentadecanoic acid (C15:0); Palmitic acid (C16:0); Palmitoleic acid (C16:1); Heptadecanoic acid (C17:0); Stearic acid (C18:0); Oleic acid (C18:1n9); Linoleic acid (C18:2n6); α-Linolenic acid (C18:3n3); Arachidic acid (C20:0); Eicosenoic acid (C20:1); Eicosadienoic acid (C20:2). SFA-Saturated fatty acids; MUFA-Monounsaturated fatty acids; PUFA-Polyunsaturated fatty acids.

**Table 2:** Phenolic compounds quantification in *Prosopis pallida* flour derived from pod mesocarp (mean ± SD).

Peak	Rt (min)	λ_max_ (nm)	Molecular Ion [M-H]^−^ (*m*/*z*)	MS^2^ (*m*/*z*)	Tentative Identification	Content [µm/g Extract]	References
**1**	9.6	333	593	503 (27), 473 (100), 443 (83), 383 (12), 353 (21), 325 (5)	Apigenin-6,8-di-*C*-hexoside (vicenin) ^A^	1864 ± 38	[31,32]
**2**	11.1	335	563	545 (23), 503 (53), 473 (100), 443 (69), 383 (39), 353 (42)	Apigenin-6-*C*-arabinoside-8-*C*-glucoside isomer 1 (Isoschaftoside) ^A^	115 ± 2	[31,32]
**3**	13.4	335	563	545 (10), 503 (78), 473 (100), 443 (79), 383 (31), 353 (28)	Apigenin-8-*C*-arabinoside-6-*C*-glucoside isomer 1 (schaftoside) ^A^	479 ± 7	[31,32]
**4**	13.7	335	563	545 (20), 503 (47), 473 (100), 443 (73), 383 (33), 353 (38)	Apigenin-6-*C*-arabinoside-8-*C*-glucoside isomer 2 (Isoschaftoside) ^A^	879 ± 22	[31,32]
**5**	15.1	330	563	545 (8),503 (73), 473 (100), 443 (83), 383 (37), 353 (26)	Apigenin-8-*C*-arabinoside-6-*C*-glucoside isomer 2 (schaftoside) ^A^	38 ± 1	[31,32]
**6**	21.3	332	623	315 (100)	Isorhamnetin-3-*O*-rutinoside ^B^	28.7 ± 0.2	DAD, MS standard
					^1^ TPC	3404 ± 52	

^1^ TPC: Total phenolic compounds. Calibration curves: A—apigenin-6-*C*-glucoside (y = 107025x + 61531, *R*^2^ = 0.9989); B—quercetin-3-*O*-glucoside (y = 34843x – 160173, *R*^2^ = 0.9987).

**Table 3:** Antioxidant, cytotoxicity, hepatotoxicity, and antimicrobial activities of *Prosopis pallida* flour derived from pod mesocarp (mean ± SD).

Bioactivities								
**Antioxidant Activity EC_50_ Values ^1^** (µg/mL)		**Antimicrobial Values (mg/mL)**						
OxHLIA			**Mesquite**		**Streptomycin**		**Ampicillin**	
Δt = 60 min	100 ± 5	**Gram negative bacteria**	**MIC**	**MBC**	**MIC**	**MBC**	**MIC**	**MBC**
Δt = 120 min	233 ± 5	*Escherichia coli*	0.20	0.30	0.20	0.30	0.40	0.50
TBARS	470 ± 6	*Enterobacter cloacae*	0.30	0.60	0.20	0.30	0.25	0.50
**Cytotoxicity GI_50_ Values^2^** (μg/mL)		*Salmonella* Typhimurium	0.20	0.30	0.20	0.30	0.75	1.20
MCF-7 (breast carcinoma)	>400	**Gram positive bacteria**						
NCI-H460 (non-small cell lung carcinoma)	242 ± 4	*Staphylococcus aureus*	0.60	0.80	0.04	0.10	0.25	0.45
HeLa (cervical carcinoma)	>400	*Listeria monocytogenes*	0.40	0.60	0.20	0.30	0.40	0.50
HepG2 (hepatocellular carcinoma)	>400	**Antifungal Values (mg/mL)**						
**Hepatotoxicity GI_50_ Values ^2^** (μg/mL)			**Mesquite**		**Ketoconazole**		**Bifonazole**	
PLP2	>400		**MIC**	**MFC**	**MIC**	**MFC**	**MIC**	**MFC**
		*Aspergillus fumigatus*	n.a	n.a	0.25	0.50	0.15	0.20
		*Aspergillus ochraceus*	0.15	0.30	0.20	0.50	0.10	0.20
		*Aspergillus niger*	n.a	n.a	0.20	0.50	0.15	0.20
		*Penicillium ochrochloron*	0.075	0.15	2.5	3.5	0.20	0.25
		*Penicillium funiculosum*	0.15	0.30	0.20	0.50	0.20	0.25
		*P. verrucosum var. cyclopium*	0.30	0.80	0.20	0.30	0.10	0.20

^1^ EC_50_ values correspond to the sample concentration achieving 50% of the antioxidant activity or 0.5 of absorbance in reducing power assay. ^2^ GI_50_ values correspond to the sample concentration achieving 50% of growth inhibition in human tumor cell lines or liver primary culture PLP2. Trolox EC_50_ values: 5.8 ± 0.6 (TBARS); OxHLIA 85 ± 2 (Δt = 60 min) and 183 ± 4 (Δt = 120 min). GI_50_ values: 1.21 mg/mL (MCF-7); 1.03 mg/mL (NCI-H460); 0.91 mg/mL (HeLa); 1.10 mg/mL (HepG2) and 2.29 mg/mL (PLP2). n.a not activity.

## Appendix A

**Table A1:** Combined effect of mesquite flour substitution level and wheat flour type on the properties of extension and stickiness of composite doughs, showing means and 95% confidence interval.

Test	Source	Extensibility (mm)			Resistance to Extension (g)					
		Mean	95% CI	Pr(>F)	Mean	95% CI	Pr(>F)			
Dough	Mesquite (%)			<0.0001			0.01			
extension	0	35.4^a^	[34.7–36.1]		43.2^a^	[41.5–44.9]				
	5	28.6^b^	[27.8–29.4]		60.6^b^	[58.8–62.4]				
	10	26.9^c^	[26.2–27.5]		65.9^c^	[64.3–67.6]				
	15	23.6^d^	[22.9–24.3]		66.3^c^	[64.8–67.9]				
	Flour type			<0.0001			<0.0001			
	55	29.0^a^	[28.4–29.5]		57.6^b^	[56.4–58.9]				
	65	28.3^b^	[27.8–28.8]		60.4^a^	[59.3–61.5]				
	Interaction			<0.0001			<0.0001			
		**Stickiness (g)**			**Work of adhesion (g⸳s)**			**Strength (mm)**		
		Mean	95% CI	Pr(>F)	Mean	95% CI	Pr(>F)	Mean	95% CI	Pr(>F)
Dough	Mesquite (%)			0.017			<0.0001			<0.0001
stickiness	0	49.0^ab^	[47.8–50.2]		4.50^c^	[4.33–4.68]		1.599^c^	[1.534–1.660]	
	5	49.4^b^	[48.1–50.6]		3.19^b^	[3.03–3.36]		1.198^b^	[1.145–1.250]	
	10	46.7^a^	[45.5–47.9]		2.49^a^	[2.34–2.65]		0.963^a^	[0.913–1.010]	
	15	47.1^ab^	[45.9–48.3]		2.52^a^	[2.36–2.69]		0.976^a^	[0.923–1.030]	
	Flour type			<0.0001			<0.0001			<0.0001
	55	52.8^a^	[52.0–53.7]		4.04^a^	[3.92–4.15]		1.445^a^	[1.404–1.486]	
	65	43.2^b^	[42.4–44.1]		2.32^b^	[2.20–2.44]		0.922^b^	[0.885–0.959]	
	Interaction			<0.0001			<0.0001			<0.0001

^a^, ^b^ and ^c^ Means followed by different superscript letters indicate significant differences according to Tukey’s test (α = 0.05).

**Table A2:** Combined effect of mesquite flour substitution level and wheat flour type on physicochemical properties of composite breads, showing means and 95% confidence intervals.

Source	Specific Volume (ml/g)			Baking Loss (%)			Water Activity (-)		
	Mean	95% CI	Pr(>F)	Mean	95% CI	Pr(>F)	Mean	95% CI	Pr(>F)
Mesquite (%)			<0.0001			<0.0001			<0.0001
0	2.29^a^	[2.27–2.32]		16.8^a^	[16.5–17.0]		0.9746^d^	[0.9743–0.9749]	
5	2.43^b^	[2.41–2.46]		17.3^b^	[17.1–17.5]		0.9726^c^	[0.9723–0.9729]	
10	2.49^c^	[2.47–2.51]		18.2^b^	[17.9–18.4]		0.9698^b^	[0.9696–0.9701]	
15	2.40^b^	[2.37–2.43]		16.6^a^	[16.3–16.8]		0.9688^a^	[0.9686–0.9691]	
Flour type			<0.0001			<0.0001			0.001
55	2.45^b^	[2.44–2.47]		17.5^a^	[17.3–17.6]		0.9717^a^	[0.9715–0.9720]	
65	2.36^a^	[2.34–2.37]		16.9^b^	[16.8–17.1]		0.9712^b^	[0.9710–0.9714]	
Interaction			<0.0001			<0.0001			<0.0001

^a^, ^b^ and ^c^ Means followed by different superscript letters indicate significant differences according to Tukey’s test (α = 0.05).

**Table A3:** Combined effect of mesquite flour substitution level and wheat flour type on the texture profile analysis descriptors of composite bread crumb, showing means and 95% confidence intervals.

Source	Hardness (g)				Springiness (-)							
	Mean		95% CI	Pr(>F)	Mean	95% CI	Pr(>F)					
Mesquite (%)				<0.0001			<0.0001					
0	1894^b^		[1834–1955]		0.971^c^	[0.966–0.976]						
5	1371^a^		[1310–1432]		0.980^c^	[0.975–0.985]						
10	1342^a^		[1278–1405]		0.963^b^	[0.958–0.969]						
15	1919^b^		[1855–1982]		0.939^a^	[0.933–0.944]						
Flour type				<0.0001			<0.0001					
55	1283^b^		[1239–1327]		0.972^a^	[0.968–0.976]						
65	1979^a^		[1935–2023]		0.955^b^	[0.951–0.958]						
Interaction				<0.0001			<0.0001					
	**Cohesiveness (-)**				**Chewiness (g)**			**Resilience (-)**				
	Mean	95% CI		Pr(>F)	Mean	95% CI	Pr(>F)	Mean		95% CI		Pr(>F)
Mesquite (%)				<0.0001			<0.0001					<0.0001
0	0.691^c^	[0.681–0.701]			1502^c^	[1457–1548]		0.316^d^		[0.311–0.320]		
5	0.677^c^	[0.666–0.688]			1035^b^	[992–1079]		0.283^c^		[0.279–0.288]		
10	0.654^b^	[0.644–0.664]			884^a^	[845–923]		0.256^b^		[0.252–0.259]		
15	0.618^a^	[0.608–0.629]			1100^b^	[1053–1148]		0.239^a^		[0.235–0.243]		
Flour type				0.455			<0.0001					<0.0001
55	0.662^a^	[0.655–0.669]			999^a^	[968–1031]		0.277^b^		[0.274–0.280]		
65	0.658^a^	[0.651–0.666]			1262^b^	[1231–1293]		0.269^a^		[0.267–0.272]		
Interaction				0.002			<0.0001					<0.0001

^a^, ^b^ and ^c^ Means followed by different superscript letters indicate significant differences according to Tukey’s test (α = 0.05).

**Table A4:** Combined effect of mesquite flour substitution level and wheat flour type on image analysis crumb features of composite bread, showing means and 95% confidence intervals.

Source	Mean Cell Area (mm^2^)			Mean Cell Density (#/mm^2^)			Cell Size Uniformity (-)		
	Mean	95% CI	Pr(>F)	Mean	95% CI	Pr(>F)	Mean	95% CI	Pr(>F)
Mesquite (%)			<0.0001			<0.0001			<0.0001
0	2.00^c^	[1.93–2.07]		0.209^a^	[0.193–0.225]		8.600^a^	[7.590–9.600]	
5	1.69^b^	[1.62–1.77]		0.249^b^	[0.234–0.265]		10.10^a^	[9.040–11.10]	
10	1.24^a^	[1.17–1.31]		0.331^c^	[0.315–0.346]		17.70^c^	[16.64–18.70]	
15	1.27^a^	[1.19–1.34]		0.316^c^	[0.300–0.332]		15.20^b^	[14.17–16.20]	
Flour type			<0.0001			<0.0001			<0.0001
55	1.75^b^	[1.70–1.80]		0.251^a^	[0.240–0.262]		10.50^a^	[9.820–11.20]	
65	1.35^a^	[1.29–1.40]		0.301^b^	[0.290–0.312]		15.21^b^	[14.49–15.90]	
Interaction			<0.0001			<0.0001			<0.0001
	**Void fraction (-)**			**Mean cell compactness (-)**			**Mean cell aspect ratio (-)**		
	Mean	95% CI	Pr(>F)	Mean	95% CI	Pr(>F)	Mean	95% CI	Pr(>F)
Mesquite (%)			<0.0001			0.156			0.383
0	0.408^b^	[0.404–0.412]		0.775^a^	[0.771–0.778]		1.61^a^	[1.59–1.62]	
5	0.410^b^	[0.406–0.414]		0.776^a^	[0.773–0.780]		1.60^a^	[1.58–1.61]	
10	0.395^a^	[0.391–0.399]		0.776^a^	[0.773–0.779]		1.60^a^	[1.58–1.62]	
15	0.397^a^	[0.393–0.401]		0.773^a^	[0.769–0.776]		1.61^a^	[1.59–1.62]	
Flour type			<0.0001			0.003			0.002
55	0.413^b^	[0.410–0.416]		0.773^a^	[0.770–0.776]		1.61^b^	[1.60–1.63]	
65	0.392^a^	[0.389–0.394]		0.777^b^	[0.775–0.780]		1.59^a^	[1.58–1.60]	
Interaction			<0.0001			0.154			0.319

^a^, ^b^ and ^c^ Means followed by different superscript letters indicate significant differences according to Tukey’s test (α = 0.05).
